# The Effects of Combining Cancer Drugs with Compounds Isolated from *Combretum zeyheri* Sond. and *Combretum platypetalum* Welw. ex M.A. Lawson (Combretaceae) on the Viability of Jurkat T Cells and HL-60 Cells

**DOI:** 10.1155/2021/6049728

**Published:** 2021-01-18

**Authors:** Morris Wende, Simbarashe Sithole, Godloves Fru Chi, Marc Y. Stevens, Stanley Mukanganyama

**Affiliations:** ^1^School of Pharmacy, College of Health Sciences, University of Zimbabwe, Mt. Pleasant, Harare, Zimbabwe; ^2^Department of Biochemistry, University of Zimbabwe, Mt. Pleasant, Harare, Zimbabwe; ^3^Department of Organic Chemistry, University of Yaoundé 1, P.O. Box 812, Yaoundé, Cameroon; ^4^Organic Pharmaceutical Chemistry, Department of Medicinal Chemistry, Uppsala Biomedical Center, Uppsala University, PO Box 574, SE-751 23 Uppsala, Sweden

## Abstract

*Combretum zeyheri* and *Combretum platypetalum* have been shown to have anticancer, antibacterial, antituberculosis, and antifungal effects in both *in vivo* and *in vitro* studies. This study sought to evaluate the antiproliferative effects of compounds isolated from *C. zeyheri* and *C. platypetalum* on Jurkat T and HL-60 cancer cell lines in combination with doxorubicin and/or chlorambucil. At their GI_50_ concentrations, the isolated compounds were combined with the corresponding GI_50_ of chlorambucil and doxorubicin. The cytotoxic effects of the combined compounds were determined on BALB/c mouse peritoneal cells. All the 4 isolated compounds had significant cytotoxic effects on Jurkat T cells. Compounds CP 404 (1), CP 409 (2), CZ 453 (3), and CZ 455 (4) had GI_50_s on Jurkat T cells of 3.98, 19.33, 6.82, and 20.28 *μ*g/ml, respectively. CP 404 (1), CP 409 (2), CZ 453 (3), and CZ 455 (4) showed GI_50_s of 14.18, 28.69, 29.87, and 16.46 *μ*g/ml on HL-60 cancer cell lines, respectively. The most potent combination against Jurkat T cells was found to be CP 404 (1) and chlorambucil. This combination showed no cytotoxic effects when tested on BALB/c mouse peritoneal cells. It was concluded that the compounds extracted from *C. zeyheri* and *C. platypetalum* inhibit the growth of Jurkat T cells *in vitro*. The combination of the compounds with anticancer drugs enhanced their anticancer effects. The combination of CP 404 (1) and chlorambucil was found not to be toxic to normal mammalian cells. Therefore, CP 404 (1), 3-*O*-*β*-L-rrhamnopyranosyl-5,7,3′4′,5′-pentahydroxyflavone, has the potential to be a source of lead compounds that can be developed for anticancer therapy. Further structure-activity relationship studies on this compound are warranted.

## 1. Introduction

Noncommunicable diseases are an increasing health problem in both developed and developing countries [1]. WHO estimates that cancer is the leading cause of death from noncommunicable diseases, followed by ischemic heart diseases, and lower respiratory tract infections and stroke [2]. Generally, cancer in developed countries is usually a result of lifestyle changes such as obesity, alcohol intake, smoking, and physical inactivity [3]. Despite having a high prevalence of cancers, African countries are not prepared to deal with this burden as they have to fight other diseases such as the human immunodeficiency virus, tuberculosis, and malaria which are also possessing a real health challenge [1, 4, 5]. It is estimated that about 6-7% of new cancer cases are diagnosed every year worldwide [6].

The antioxidant defence mechanism causes cell damage from free radicals (OFR) which is ubiquitous. The OFR-associated lesions that will not result in cell death can stimulate the development of cancer [7, 8]. Endogenous DNA lesions left behind are genotoxic and can induce mutations. Oxygen radicals have been shown to oxidise lipids or proteins, thus generating intermediates that react with DNA forming adducts. Reactive Oxygen Species (ROS) have been observed to facilitate cancer cell proliferation and increase the rate of survival and adaptation to hypoxia [9]. An increase in proliferation of the blood cells in the bone marrow and blood-producing organs leads to a condition called leukaemia [10]. Progression of leukaemia may be sudden (acute) or slow gradual (chronic). Acute and chronic leukaemia mainly occurs in children and adults, respectively. Generally, a malignancy that involves myeloid cells, granulocytes (neutrophils, eosinophil, and basophils), and macrophages leads to myeloid leukaemia, and that which involves T and B lymphocytes is called lymphocytic leukaemia [11].

Drugs used in the management of cancer usually inhibit the growth of tumour cells or kill the tumour cells [12]. There are several classes of anticancer compounds that include: alkylating agents, antimetabolites, cytotoxic antibiotics, plant derivatives, and protein kinase inhibitors [13]. Chemotherapy usually combines several drugs to provide a large cytoreduction in several tumourcells. Various methods such as dose reduction, cytoprotective agents, alternative drugs, or their analogues such as growth factors are used to decrease the incidences of adverse effects [14].

Modern health care practice is also slowly embracing the use of traditional and herbal medicines in the management of various ailments. *Combretum zeyheri* is known by the local community with various names such as large-fruited Bushwillow [15]. The use of *C. zeyheri* has been documented for treating many diseases in both South and Eastern Africa. The plant has primarily been used in the management of hookworm infections, dysentery, coughs, vomiting, sore throat, stomachache, and diarrhoea [16]. *Combretum platypetalum* is a shrub that is less than 30 cm that grows from woody root state and is distributed in Africa in countries such as DRC, Tanzania, Mozambique, Zambia, and Zimbabwe. The shrub has been used in traditional medical practice to manage swelling which is caused by mumps as well as pneumonia, diarrhoea, and dysmenorrhea [17]. The extract of *C. platypetalum* is cytotoxic to cancer cell lines *in vitro* [18]. Some compounds from the Combretaceae species have been shown to have anticancer activity [19].

Anticancer drugs used in chemotherapy often fail to treat cancers resulting in unresponsiveness to chemotherapy or recurrence of secondary cancers. For successful cancer treatment, sometimes two drugs are used. The development of drug resistance during chemotherapy decreases the success of chemotherapy [20]. Plants are good sources of compounds that are active as anticancer agents or that can be used as leads to the discovery of new anticancer drugs [21]. This study was set up to investigate the combination of drugs used in the management of cancer with phytochemical constituents from the Combretaceae species. This study provided valuable information about synergistic or antagonistic effects of taking herbal medicines together with conventional drugs. The aim of the study was to investige the anticancer activity of the compounds isolated from *C. zeyheri* and *C. platypetalum* on Jurkat T cells and HL-60 cells when used singly and when combined with chlorambucil and doxorubicin.

## 2. Materials and Methods

The chemicals and the cancer cells used in the study were obtained from Sigma-Aldrich Chemicals Company (Munich, Germany). The digital light microscope from Celestron (Los Angeles, USA) and haemocytometer were used to count cells. Incubations were carried out in a CO_2_ SHEL LAB incubator (Sheldon Mfg. Inc., Cornelius, USA).Analyses of cell density was done using a Tecan Microplate reader (Tecan Group Ltd., Männedorf, Switzerland). The leaves of *C. zeyheri* and *C. platypetalum* plants were collected in Norton, Mashonaland Central province, Zimbabwe, whose geographical location is 17.8833°S, 30.7000°E, 1364 m above sea level. The plan sample identity was authenticated by Mr. Christopher Chapano, a taxonomist at the National Botanic Gardens (Harare, Zimbabwe). Voucher specimen Nos. N6E7 and N9E7 respectively were deposited at the University of Zimbabwe, Biochemistry Department. Leaves were dried and ground into powder before extraction. A mass of 800 g of powdered *C. platypetalum* leaves or 950 g of powdered *C. zeyheri* was extracted with a DCM : MeOH mixture (1 : 1 ratio). The DCM : MeOH extract was concentrated under reduced pressure to yield a dark green residue and then submitted to a silica gel (200–300 mesh) column and eluted with mixtures of n-hexane, EtOAc, and MeOH of increasing polarity, giving fractions. C. platypetalum was subjected to column chromatography over silica gel 230–400 mesh (Merck), eluted with a gradient of n-hexane (100-0%)/EtOAc mixtures (0-100%) and finally 10% Methanol), and yielded compounds CP 409, CP 404 whilst the *C. zeyheri* extract yielded compounds Cz 453 and Cz 455.

### 2.1. Identification of Isolated Compounds

The isolated compounds were identified based on their NMR and MS data in corroboration with data from the literature. ^1^H NMR spectra were recorded at 400 MHz and ^13^C NMR spectra at 100 MHz. The chemical shifts for ^1^H NMR and ^13^C NMR were referenced to TMS via residual solvent signals (^1^H, CDC^l3^ at 7.26 ppm; ^13^C, CDCl_3_ at 77.36 ppm; ^1^H, DMSO-d6 at 2.45 ppm; ^13^C, DMSO-d6 at 39.43 ppm, ^1^H, CD_3_OD at 3.31 ppm; ^13^C, CD_3_OD at 49.0 ppm). 2D NMR experiments were run using standard pulse sequences. Molecular formulae were determined by electrospray ionization with a 7-T hybrid ion trap and a TOF detector running in positive or negative mode.

### 2.2. Cytotoxicity Effects of Natural Compounds and Standard Anticancer Drugs on Jurkat T and HL-60 Cells

Compounds used in this study were named as follows: CP 409, CP 404, CZ 455, and CZ 453; these were dissolved in dimethyl sulfoxide and RPMI 1640 media to make final concentrations of 25 *μ*g/ml, 12.5 *μ*g/ml, and 6.5 *μ*g/ml. The two anticancer drugs, chlorambucil and doxorubicin, were dissolved in DMSO and RMPI 1640 to make concentrations of 5 *μ*g/ml, 2 *μ*g/ml, and 1 *μ*g/ml. Cytotoxicity of the natural compounds and anticancer drugs was determined for both Jurkat T and HL-60 cells in 12-well plates using the Trypan blue exclusion assay. After seeding all the reagents, the plates were incubated for 72 hours at 37°C and 5% carbon dioxide in a humidified incubator. The negative control consisted of the cells media only. The positive control chlorambucil was used at 10 *μ*g/ml, and doxorubicin was used at 10 *μ*g/ml. GraphPad Prism 6 was used to determine the GI_50_ of the compounds exposed to both Jurkat T cells and HL-60 cancer cell lines.

### 2.3. Cytotoxic Effects of the Combination of the Anticancer Drugs and Phytochemical Constituents

The MTT (3-(4, 5-dimethylthiazol-2-yl)-2, 5-diphenyltetrazolium bromide) assay was used to measure the effects of combining the anticancer drugs (doxorubicin and chlorambucil) with the phytochemical constituents from *C. zeyheri* and *C. platypetalum*. From the GI_50_ of the compounds and anticancer drugs, the following concentrations were prepared: GI_50_, 1/2 GI_50_, 1/4 GI_50_, 1/8 GI_50_, 1/16 GI_50_, and 1/32 GI_50_ in *μ*g/ml. The compounds were added in equal volumes of 10 *μ*l each. The positive control used for the combination assay involving chlorambucil was doxorubicin at 10 *μ*g/ml and that of doxorubicin was chlorambucil at 10 *μ*g/ml. After adding all the reagents, the 96-well microplates were incubated for 72 hours at 37°C in a 5% carbon dioxide humidified SHEL-LAB incubator (Sheldon Mfg. Inc., Cornelius, USA). Absorbance was measured at 590 nm using a Tecan Microplate reader (Tecan Group Ltd., Männedorf, Switzerland).

### 2.4. Toxicological Effects of Combing Anticancer Drugs and Phytochemical Constituents on Mouse Peritoneal Cells

The induction and harvesting procedure for obtaining viable immune cells from the peritoneal cavity of mice was carried out as described by Ray and Dittel [22]. Toxicological evaluation of the effects of combined compounds was carried out by the combination of CP 404 with chlorambucil. The GI_50_ concentrations of CP 404 were combined with a GI_50_ of chlorambucil. RPMI media, compounds (anticancer compounds and phytochemicals), and mouse peritoneal cells were seeded into 12-well plates and incubated for 72 hours. Cell counts were then performed using the Trypan blue exclusion assay.

### 2.5. Statistical Analyses

Graphical and statistical analyses were carried out using GraphPad Prism version 6. The data were expressed in the form of mean ± standard deviation of the mean. Significant differences between various means of controls and the tests were analyzed using the one-way ANOVA using Dunnett's Multiple Comparison Post Test with a *P* value of 0.05.

## 3. Results and Discussion

Cancer is a debilitating disease that claimed an estimated 9.6 million deaths in 2018 [2]. Drug combinations may used in the management of cancer as there is a high diversity of tumour malignancy and incomplete use of treatment regimen that encompasses one or more modalities which essentially leads to drug resistance [23, 24]. The mechanism of anticancer drugs is usually different, and a combination of anticancer drugs leads to the cure of cancer or to considerably prolonged life as these combinations present multidrug pathway targets leading to improved therapy [25, 26]. Drug combinations also help in reducing the adverse effects of anticancer drugs, thereby, improving the patient's quality of life. Anthracyclines such as doxorubicin and biodioxopiperazine such as razoxane have been combined leading to decreased drug resistance and neoplasm metastasis [24].

### 3.1. Spectroscopic Identification of Isolated Compounds

Spectrometric analysis of the pure compounds CZ 453 and CZ 455 revealed that the compounds were waxes. However, CP 404 and 409 were found to be pure compounds. Outlined below is the data for the compound CP 404 (1):

*Compound 1 (CP 404)*: myricetin-3-O-glucoside or 3-*O*-*β*-D-glucopyranosyl-3′,4′,5′,5,7-pentahydroxyflavone, amber powder; HR-ESI-MS *m/z* 481.0986 for [M+H]^+^ (calcd 481.0982) corresponding to C_21_H_20_O_13_H, PubChem CID: 18604930.

^1^H NMR (500 MHz, MeOD) *δ*(ppm): 6.94 (1H, s, H-2′), 6.93 (1H, s, H-6′), 6.36 (1H, d, 2.5 Hz, H-8), and 6.20 (1H, d, 2.5 Hz, H-6); glucosyl: 5.31 (1H, d, 8.0 Hz, H-1^″^), 3.81 (1H, dd, 9.5, 3.6 Hz, H-6^″^a), 3.79 (1H, dd, 12.0, 2.0 Hz, H-6^″^b), 3.53 (1H, dd, 7.5, 4.5 Hz, H-5^″^), 3.47 (1H, t, 12.0 Hz, H-5^″^), and 3.24 (1H, t, 2.5 Hz, H-4^″^). ^13^C NMR (125 MHz, MeOD) *δ*(ppm): 177.9 (C-4), 164.5 (C-7), 164.4 (C-5), 161.1 (C-2), 157.9 (C-9), 145.4 (C-3′), 144.9 (C-5′), 136.6 (C-4′), 134.8 (C-3), 108.8 (C-2′), 108.3 (C-6′), 102.9 (C-10), 98.4 (C-6), and 93.1 (C-8); glucosyl: 103.3 (C-1^″^), 76.9 (C-5^″^), 76.7(C-2^″^), 74.2 (C-3^″^), 70.4 (C-4^″^), and 61.2 (C-6^″^).

*Compound 2 (CP 409)*: myricitrin or myrecitrin-3-O-rhamnoside, 3-*O*-*α*-L-rhamnopyranosyl-3′,4′,5′,5,7-pentahydroxyflavone, yellow powder; HR-ESI-MS *m/z* 466.1036 for [M+2H]^+^ (calcd 466.1111) corresponding to C_21_H_20_O_12_2H, PubChem CID: 5281673. ^1^H NMR (500 MHz, MeOD) *δ*(ppm): 6.94 (2H, s H-2′,6′), 6.36 (1H, d, 2.5 Hz, H-8), and 6.20 (1H, d, 2.5 Hz, H-6); rhamnosyl: 5.31 (1H, d, 2.0 Hz, H-1^″^), 4.22 (1H, dd, 4.0, 2.0 Hz, H-2^″^), 3.78 (1H, dd, 12.0, 4.0 Hz, H-3^″^), 3.52 (1H, dd, 7.5, 4.5 Hz, H-5^″^), 3.36 (1H, t, 12.0 Hz), and 0.96 (3H, d, 8.0 Hz, H-6′). ^13^C NMR (125 MHz, MeOD) *δ*(ppm): 178.2 (C-4), 164.4 (C-7), 164.3 (C-5), 161.7 (C-2), 157.0 (C-9), 145.4 (C-3′), 145.4 (C-5′), 138.6 (C-4′), 136.2 (C-3), 108.1 (C-2′,6′), 102.2 (C-10), 98.6 (C-6), and 93.2 (C-8); rhamnosyl: 103.2 (C-1^″^), 71.9 (C-2^″^), 70.7 (C-3^″^), 70.6 (C-4^″^), 70.4 (C-5^″^), and 16.2 (C-6^″^).

**Figure 1:**
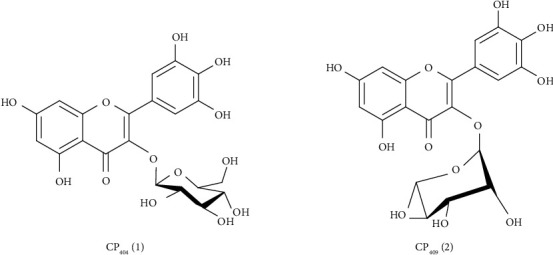
Structures of compounds isolated from *Combretum platypetalum*. CP 404 (1): 3-*O*-(*β*-D-glucopyranosyl)-3′4′,5′,5,7-pentahydroxyflavone; CP 409: 3-O-(*β*-L-rhamnopyranosyl)-3',4',5',5,7-pentahydroxyflavone.

Spectrometric analysis of the compound showed that CP 409 was 3-O-(*α*-L-rhamnopyranosyl)-3′,4′,5′,5,7-pentahydroxyflavone, and the structure is shown in Figure 1(a). The analysis of the NMR data showed that CP 404 was 3-*O*-(*β*-D-glucopyranosyl)-3,4′,5′5,7-pentahydroxyflavone, and the structure is shown in Figure 1(b). These were in agreement with data in the literature for these compounds [27, 28].

### 3.2. Antiproliferative Effects of Compounds on Cancer Cell Lines

**Figure 2:**
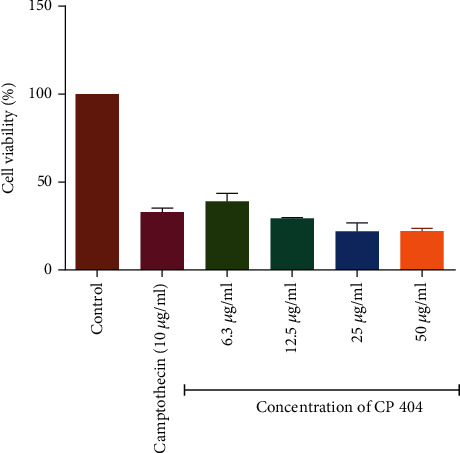
Shows the antiproliferative effects of CP 404 on Jurkat T cells after incubation for 72 hours. The percentage of cell viability was compared to cells with cell media only. The results are showing that as the concentration increases, there were statistical significance decreases in cell viability. Compound 1 had a GI_50_ value of 3.98 *μ*g/ml. The values are mean ± standard deviation (SD) of 4 replicate data sets. Statistical one-way ANOVA (^∗∗∗^*P* < 0.0001).

The Trypan blue exclusion assay was used to determine the cell viability of Jurkat T and HL-60 cells when incubated with four compounds isolated from *C. zeyheri* and *C. platypetalum*. Compounds isolated from *C. platypetalum* inhibited the growth of Jurkat T cells with both compounds showing a dose-dependent antiproliferative and cytotoxic inhibition of growth on Jurkat T cells. Of these compounds, CP 404 presented the most potent reduction of cell viability of Jurkat T cells having a GI_50_ of 28.7 *μ*g/ml (Table 1). Chlorambucil had GI_50_ value of 2.99 *μ*g/ml while that of doxorubicin was 2.50 *μ*g/ml on Jurkat T cells (Table 1). CP 404 showed a similar profile in terms of reduction of cell viability from the lowest concentration of 6.3 *μ*g/ml to the highest concentration of 50 *μ*g/ml (Figure 2). The cell viability was decreased by 64% after treatment with 6.3 *μ*g/ml CP 404 for 72 hours and further reduced by up to 79% with a higher concentration (50 *μ*g/ml) of the compound. Compounds isolated from *C. zeyheri* also possessed antiproliferative effects and cytotoxic activity against Jurkat T cells. The compound CZ 453 had a higher inhibitory effect against Jurkat T cells than CZ 455 though both compounds had dose-dependent antiproliferative activity.

**Figure 3:**
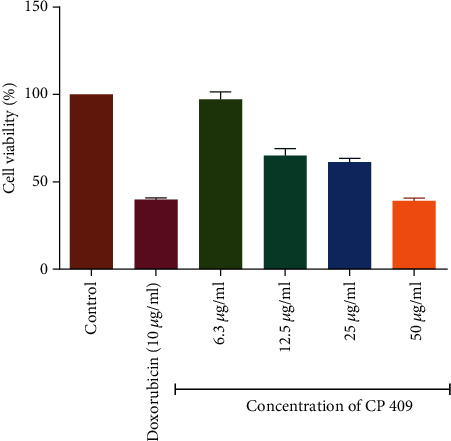
The antiproliferative effects of CP 409 on HL-60 cancer cell lines. The graph shows that as the concentration increases, there were statistical significance decreases in cell viability. The percentage of cell viability was compared to cells with cell media only. The values are mean ± standard deviation (SD) of 4 replicate data sets. Statistical one-way ANOVA was used to analyze the results (^∗∗∗^*P* < 0.001).

**Figure 4:**
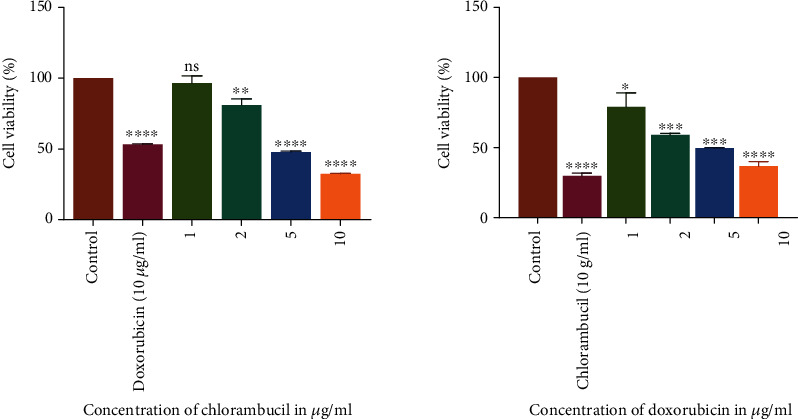
The antiproliferative effects of chlorambucil and doxorubicin on Jurkat T cancer cell lines. The graphs show that as the concentration increases, there was a statistically significant decrease in cell viability compared to the cell with cell media only. The values are mean ± standard deviation (SD) of 2 replicate data sets. Statistical one way ANOVA was used to analyze the results (^∗∗∗^*P* < 0.001).

**Figure 5:**
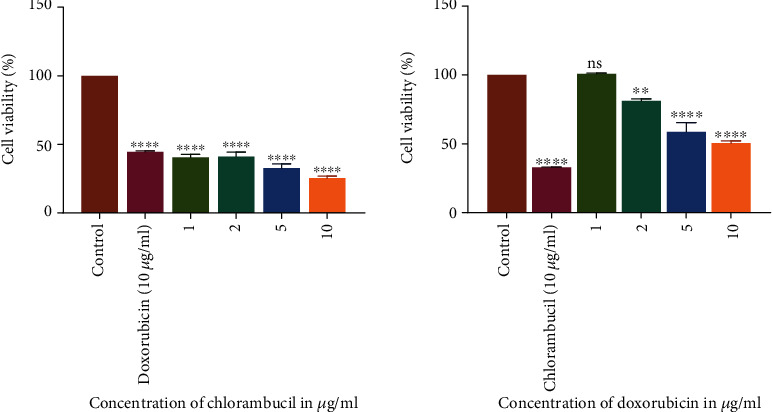
The antiproliferative effects of chlorambucil and doxorubicin on HL-60 cancer cell lines. The graphs show that as the concentration increases, there was a statistically significant decrease in cell viability compared to the cell with cell media only. The values are mean ± standard deviation (SD) of 2 replicate data sets. Statistical one way ANOVA was used to analyze the results (^∗∗∗^*P* < 0.001).

The results summarized in Table 2 showed that all the compounds, as well as standard anticancer drugs incubated with HL-60 cells, exhibited a significant inhibitory effect on the proliferation of these cells. The GI_50_ values for chlorambucil and doxorubicin were 1.79 and 3.87 *μ*g/ml on HL-60 cells, respectively (Table 2). The cell viability declined in a concentration-related manner for all the compounds with CP 409 (Figure 3) showing the greatest potency with a GI_50_ value of 14.2 *μ*g/ml, though it was not comparable to the positive controls. The flavonoid compound from *C. platypetalum*, CP 409, had a dose-dependent cytotoxic and antiproliferative effects on HL-60. Compound CP 404, however, possessed the most significant antiproliferative effects at the highest concentration used in this study, and these results are consistent with previous findings [29, 30]. Doxorubicin showed concentration-dependent effects in suppressing the growth of Jurkat T and HL-60 cells *in vitro*. Comparing with the negative control, that is cells with media only, reduction in the number of viable Jurkat T cells occurred at a concentration of 2 *μ*g/ml (Figure 4). Chlorambucil antiproliferative effects were evaluated *in vitro* on Jurkat T and HL-60 cells; there was concentration-dependent inhibition of growth of HL-60 cells (Figure 5).

Plants have been a source of bioactive compounds for use as anticancer drugs and source of agents which may be used as chemopreventive agents [31]. Polyphenolic compounds are a part of a large diversity of natural compounds and this group also comprise of flavonoids [32]. Epidemiological studies have revealed that consumption of plants and fruits rich in flavonoids leads to a decrease in the development of a vast array of cancers [33]. According to the National Cancer Institute (NCI), extracts isolated from plants with GI_50_ values of ≤30 *μ*g/ml are regarded as active [34]. All the compounds used in the study had a GI_50_ value of less than 30 *μ*g/ml and, hence, are regarded as active according to this criterion. High antiproliferative effects of the fraction CZ 453 may be due to the combination of various phytochemicals such as saponins, tannins, flavonoids, and triterpenes acting synergistically against Jurkat T cells and HL-60 cells [32]. Compounds extracted using methanol have been shown to have antiproliferative activity and are cytotoxic against cancer cell lines [35].

Antiproliferative activity is usually different among cancer cell lines; hence, the compounds showed different cytotoxic activity on Jurkat T cells and HL-60 cells as exhibited by different GI_50_ values [35, 36, 37]. The flavonoid drug phenoxodiol, which is undergoing clinical trials, has been shown to kill primarily the myeloid, rapidly proliferating cells, and lymphoid blast cells [30]. Decrease in cell viability by Jurkat T cells and HL-60 when treated with compound CP 409 may be attributed to the presence of flavonoids that kill the cells [38]. Flavonoids have been shown to inhibit cell proliferation through cell cycle arrest. A flavonoid baicalein has been shown to cause a decrease in levels of Cdk, cyclin D1, and cyclin B1 cell arrest in human lung carcinoma cells [39]. Methanol plant extracts have been shown to cause cell death by apoptosis and inhibition of G2/M cell cycle arrest [36, 40]. Apoptosis of the flavonoids has been shown to occur as a result of nuclear condensation, DNA fragmentation, and cell shrinkage [36].

### 3.3. Cytotoxic Effects of the Combination of the Anticancer Drugs and Phytochemical Constituents

**Figure 6:**
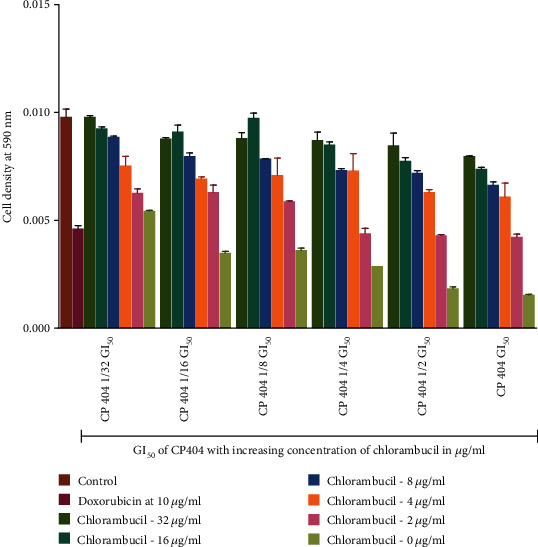
The antiproliferative effects of combining CP 404 and chlorambucil on Jurkat T cells. Chlorambucil (0–16 *μ*g/ml) and CP 404 (GI_50_, 1/2 GI_50_, 1/4 GI_50_, 1/8 GI_50_, 1/16 GI_50_, and 1/32 GI_50_) were combined and incubated with Jurkat T cells for 72 hours. The MTT assay was used to determine the number of viable cells present in the sample. When Jurkat T cells were incubated with varying amounts of chlorambucil combined with CP 404, there was no significant difference in the cell viability. Cell density corresponds to cell viability: the higher the cell density, the more viable cells present in the sample. The values are mean ± standard deviation (SD) of 2 replicate data sets.

**Figure 7:**
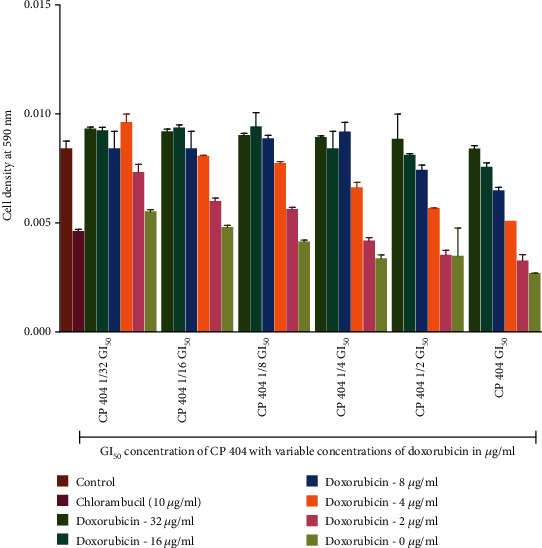
The antiproliferative effects of combining CP 404 and doxorubicin on Jurkat T cells. The antiproliferative effects of CP 404 (GI_50_, 1/2 GI_50_, 1/4 GI_50_, 1/8 GI_50_, 1/16 GI_50_, and 1/32 GI_50_), when combined with doxorubicin (0–16 *μ*g/ml), were determined using the MTT assay to investigate the number of viable cells present in the sample. The results show that at low concentration of chlorambucil, there was no significant reduction in cell density compared to cells with cell (RPMI) media only. The cell density corresponds to the number of viable cells present in the sample. The values are mean ± standard deviation (SD) of 2 replicate data sets.

The MTT (3-(4, 5-dimethylthiazol-2-yl)-2, 5-diphenyltetrazolium bromide) assay was used to determine the antiproliferative effects of the compounds when combined with two anticancer drugs, chlorambucil and doxorubicin. The investigation of antiproliferative effects of the compounds in combination with anticancer drugs used the GI_50_ concentration of the compounds and that of anticancer drugs. The investigations were carried out with Jurkat T cells, which were more susceptible to the isolated compound (CP 404) as well as anticancer drugs. The dose-response graphs, showing visual representation, as well as combination indices, giving a quantitative measure, were used to determine the antiproliferative effects of the combined drug action. At low concentration, the compounds did not cause any significant reduction in cell density. There was greater cell death with no significant reduction in cell density when CP 404 GI_50_/32 to CP 404 GI_50_/4 combined with increasing GI_50_ concentration of chlorambucil (Figure 6) and doxorubicin (Figure 7) on Jurkat T cells. The antiproliferative effect of combining standard anticancer drugs with CP 404 was calculated using the following formula [41]:

(1) $$\begin{array}{c} \text{Combination Index }\left(\text{CI}\right)=\frac{\text{inhibition of CP }404+\text{anticancer drug }}{\text{inhibition of anticancer drug}}. \end{array}$$

The CI values were then interpreted as follows: <0.5: synergism, >0.5 to 1.0: no interaction, and 1.0 to 4.0: antagonism. The combination index value showed that there was antagonistic effect (CI = 1.0 to 4.0) between CP 404 and chlorambucil as well as doxorubicin. There was, however, death of cells as shown by a decline in cell viability (Figure 7). At higher concentrations of CP 404, cell death increased; with this, it may be stated that the cell growth of Jurkat T is independent of concentrations of the anticancer drug. In this study, the combination of CP 404 chlorambucil, as well as doxorubicin, exerts an effect that is less than additive showing antagonistic effects in the drug interaction. It has been shown that the activity of a single compound may be reduced when incubated with another [42]. The cell growth of Jurkat T cells is inhibited by treating the cells with CP 404 without chlorambucil or doxorubicin. Previous studies have shown that combined drug action is greater when phytochemicals are added first rather than the converse [43].

Studies have shown that a combination of natural compounds with anticancer drugs increases their cytotoxic effect by various mechanisms such as sensitizing the cells to the drugs, and the effect depends on the dose of the combination [44]. The decrease in cell viability exhibited as a result of incubation with our compounds shows that the phytochemicals in plant fractions might have acted on various targets in the Jurkat T cells [45, 46]. A study was done by Yue et al. [47] that indicated that triterpenes in *Ganoderma lucidum*, a plant used in Traditional Chinese medical practices in the management of cancer, has an enhanced effect inhibiting proteins that cause cell proliferation, cell cycle, and apoptosis when combined with doxorubicin against HeLa cells without necessarily acting in synergy. Cytotoxicity of the compounds may be a result of phytochemicals causing the production of ROS of the anticancer drugs doxorubicin and chlorambucil [47]. A combination of extracts from *Boswellia serrata* and doxorubicin against human hepatocellular carcinoma cells demonstrated that the combination was more effective and showed dose-dependent increases in the level of TNF-*α*, IL-6, and caspase-3 activity [48]. The production of TNF-*α*, IL-6, and caspase-3 activity has been shown to cause induction of apoptosis in cancer cells.

### 3.4. Toxicological Investigations with Mouse Peritoneal Cells

Toxicological studies are usually done to predict the potential toxicity of the drug during drug development [49]. Toxicological evaluation of the combination of CP 404 and chlorambucil was carried out using mouse peritoneal cells as this combination showed the highest antiproliferative effects on Jurkat T cells. Mouse peritoneal cells or normal human cells are usually used to study the cytotoxic effects of the drugs *in vitro*. Assessment of the cytotoxic assay is usually done by observing morphology, cell viability, differentiation markers, and inhibition of cell proliferation [50]. The capacity of the plant to inhibit growth and decrease cell viability is also an indication of toxicity [51]. Toxicological evaluation of the combination of CP 404 and chlorambucil was evaluated using mouse peritoneal cells as this combination had shown the greatest potency on cancer cell lines. The Trypan blue exclusion assay was used to determine the number of viable cells present in the sample after incubating the combinations for 72 hours.

**Figure 8:**
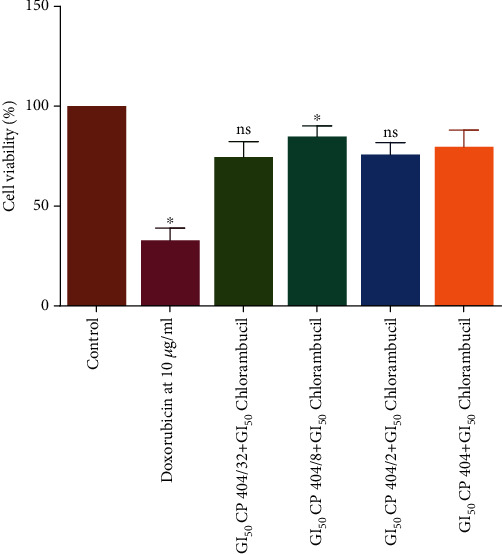
The cytotoxic effects of combining CP 404 and chlorambucil on mouse peritoneal cells. The Trypan blue exclusion assay was used to determine the number of viable cells present in the sample after incubating the combinations for 72 hours. The results show the cytotoxicity effects of a combination of CP 404 and chlorambucil on mouse peritoneal cells. The values are mean ± standard deviation of two separate experiments. Statistical one-way ANOVA was used to analyze the results (^∗^*P* < 0.05).

The results showed that the combination did not lead to a statistically significant reduction in cell viability at the highest concentration of CP 404 and chlorambucil combination used. The decrease in cell viability was, however, minimal with only about 10% difference compared to the controls (Figure 8). In general, the combination did not result in a more than 10% reduction in the number of viable mouse peritoneal cells; hence, it was considered not to be toxic because it has been shown that for a compound to be stated as toxic, it should at least exhibit a 30% decrease in cell viability [52]. Toxicity of anticancer drugs such as doxorubicin is toxic to mouse peritoneal cells by production of Toll-Like Receptor 4 (TLR4) which also causes the cells to be sensitive to endotoxins which may cause the cells to die [53]. Studies have shown that the toxicological effect of phytochemicals is usually minimal to mouse macrophages [54]. Many phytochemicals such as saponins, lectins, starch, polypeptides, and lectins are not toxic to T and B cells [55, 56].

## 4. Conclusion

The current study was designed to determine the effect of combining compounds isolated from *Combretum zeyheri* and *Combretum platypetalum* against Jurkat T and promyelocytic leukaemia HL-60 cells. The combination of chlorambucil and doxorubicin was the most potent against Jurkat cells exhibiting synergistic effects in a dose-dependent manner. Combination of phytochemicals and standard drugs resulted in a reduction in cell viability that can be attributed to effects of the isolated compounds as the effects of the anticancer drug were notably the same. The most potent combination did not reduce (by more than 10%) the cell viability of mouse peritoneal cells showing that the compounds are not toxic to normal cells. This study contributes to the understanding of the ethnopharmacological use of the plants against cancer in Southern Africa. Plant extracts and phytochemicals from *Combretum zeyheri* and *Combretum platypetalum* can, therefore, be used as lead candidates for anticancer drug development.

## Figures and Tables

**Table 1:** Summary of GI_50_ of compounds against Jurkat T cells by Trypan blue assay for various concentrations in *μ*g/ml.

Name of compound	GI_50_ (*μ*g/ml)
CP 409	19.3
CP 404	3.98
CZ 453	6.24
CZ 455	20.3
Chlorambucil	2.99
Doxorubicin	2.50

**Table 2:** Summary of GI_50_ of compounds against HL-60 cells by Trypan blue assay for various concentrations in *μ*g/ml.

Name of compound	GI_50_ (*μ*g/ml)
CP 409	14.2
CP 404	28.7
CZ 453	16.5
CZ 455	29.9
Chlorambucil	1.79
Doxorubicin	3.87

## Data Availability

The datasets used and/or analyzed during the current study are available from the corresponding author on reasonable request.

## References

[1] Parkin D. M., Bray F., Ferlay J., Jemal A. (2014). Cancer in Africa 2012. *Cancer Epidemiology and Prevention Biomarkers*.

[2] World Health Organization (2018). *World health statistics 2018: monitoring health for the SDGs sustainable development goals*.

[3] Anand P., Kunnumakara A. B., Sundaram C. (2008). Cancer is a preventable disease that requires major lifestyle changes. *Pharmaceutical Research*.

[4] Oppah K., Alice M., Nomsa T. (2017). Cervical cancer in Zimbabwe: a situation analysis. *The Pan African Medical Journal*.

[5] Olaleye O., Ekrikpo U. (2017). Epidemiology of cancers in sub-Saharan Africa. *Cancer in Sub-Saharan Africa*.

[6] Bray F., Ferlay J., Soerjomataram I., Siegel R. L., Torre L. A., Jemal A. (2018). Global cancer statistics 2018: GLOBOCAN estimates of incidence and mortality worldwide for 36 cancers in 185 countries. *CA: a Cancer Journal for Clinicians*.

[7] Valko M., Izakovic M., Mazur M., Rhodes C. J., Telser J. (2004). Role of oxygen radicals in DNA damage and cancer incidence. *Molecular and Cellular Biochemistry*.

[8] Pourahmad J., Salimi A., Seydi E. (2016). Role of oxygen free radicals in cancer development and treatment. *Free radicals and diseases*.

[9] Reczek C. R., Chandel N. S. (2015). ROS-dependent signal transduction. *Current Opinion in Cell Biology*.

[10] Roy M., Mukherjee A., Mukherjee S., Biswas J. (2017). Nutraceuticals in leukaemia. *Journal of Ayurvedic and Herbal Medicine*.

[11] Haouas H., Haouas S., Uzan G., Hafsia A. (2010). Identification of new markers discriminating between myeloid and lymphoid acute leukaemia. *Haematology*.

[12] Selzer E., Kornek G. (2013). Targeted drugs in combination with radiotherapy for the treatment of solid tumours: current state and future developments. *Expert Review of Clinical Pharmacology*.

[13] Flower R. J., Henderson G., Rang H. P., Ritter J. M. (2016). *Rang and Dale's Pharmacology*.

[14] Hoagland H. C. (1982). Hematologic complications of cancer chemotherapy. *Seminars in oncology*.

[15] Chapano C., Mugarisanwa N. (2003). *Plants of the Matobo district', National Herbarium and Botanic Garden, Zimbabwe*.

[16] Fowler A., Sommer V. (2007). Subsistence technology of Nigerian chimpanzees. *International Journal of Primatology*.

[17] Marston A., Msonthi J. D., Hostettmann K. (1996). *Polyphenolic constituents of Brackenridgea zanguebarica (Ochnaceae) and their biological activity*.

[18] Chiramba O., Mukanganyama S. (2016). Cytotoxic effects of Combretum platypetalum Welw. ex MA Lawson subsp. oatesii (Rolfe) Exell (Combretaceae) leaf extracts on Jurkat T-cells and reversal of effects by reduced glutathione. *Journal of Biologically Active Products from Nature*.

[19] El-Kashak W. A., Osman S. M., Gaara A. H., El-Toumy S. A., Mohamed T. K., Brouard I. (2017). Phenolic metabolites, biological activities, and isolated compounds of Terminalia muelleri extract. *Pharmaceutical Biology*.

[20] Donmez Y., Akhmetova L., Iseri O. D., Kars M. D., Gunduz U. (2011). Effect of MDR modulators verapamil and promethazine on gene expression levels of MDR1 and MRP1 in doxorubicin-resistant MCF-7 cells. *Cancer Chemotherapy and Pharmacology*.

[21] Heinrich M. B., Gibbons S., Williamson E. M. (2004). *Fundamentals of Pharmacognosy and Phytotherapy*.

[22] Ray A., Dittel B. N. (2010). Isolation of mouse peritoneal cavity cells. *JoVE (Journal of Visualized Experiments)*.

[23] Durmaz R., Deliorman S., Uyar R., Işiksoy S., Erol K., Tel E. (1999). The effects of anticancer drugs in combination with nimodipine and verapamil on cultured cells of glioblastoma multiforme. *Clinical Neurology and Neurosurgery*.

[24] Lu D. Y., Chen E. H., Ding J., Xu B., Lu T. R. (2015). Anticancer drug combinations, a big momentum is needed. *Metabolomics*.

[25] Druker B. J. (2003). Chronic myeloid leukaemia in the imatinib era. *Seminars in haematology*.

[26] Tipping A. J., Melo J. V. (2003). Imatinib mesylate in combination with other chemotherapeutic drugs: in vitro studies. *Seminars in haematology*.

[27] Fossen T., Larsen Å., Kiremire B. T., Andersen Ø. M. (1999). Flavonoids from blue flowers of Nymphaèa caerulea. *Phytochemistry*.

[28] Aderogba M. A., Akinkunmi E. O., Mabusela W. T. (2011). Antioxidant and antimicrobial activities of flavonoid glycosides from Dennettia tripetala G.Baker lLeaf eExtract. *Nigerian Journal of Natural Products and Medicine*.

[29] Jain R., Jain S. K. (2011). Screening of in vitro cytotoxic activity of some medicinal plants used traditionally to treat cancer in Chhattisgarh state, India. *Asian Pacific Journal of Tropical Biomedicine*.

[30] Liu X., Ye F., Wu J., How B., Li W., Zhang D. Y. (2015). Signalling proteins and pathways affected by flavonoids in leukaemia cells. *Nutrition and Cancer*.

[31] Fulda S. (2009). Tumour resistance to apoptosis. *International Journal of Cancer*.

[32] Baharum Z., Akim A. M., Taufiq-Yap Y. H., Hamid R. A., Kasran R. (2014). In vitro antioxidant and antiproliferative activities of methanolic plant part extracts of Theobroma cacao. *Molecules*.

[33] Ferguson P. J., Kurowska E., Freeman D. J., Chambers A. F., Koropatnick D. J. (2004). A flavonoid fraction from cranberry extract inhibits proliferation of human tumour cell lines. *The Journal of Nutrition*.

[34] Suffness M., Pezzuto J. M. (1990). *Assays related to cancer drug discovery. in Methods in plant biochemistry: assays for bioactivity*.

[35] Rascon Valenzuela L. A., Jimenez Estrada M., Velazquez Contreras C. A. (2015). Antiproliferative and apoptotic activities of extracts of Asclepias subulata. *Pharmaceutical Biology*.

[36] Talib W. H., Mahasneh A. M. (2010). Antiproliferative activity of plant extracts used against cancer in traditional medicine. *Scientia Pharmaceutica*.

[37] Gabrani R., Jain R., Sharma A., Sarethy I. P., Dang S., Gupta S. (2012). Antiproliferative effect of Solanum nigrum on human leukemic cell lines. *Indian Journal of Pharmaceutical Sciences*.

[38] Park H. J., Kim M. J., Ha E., Chung J. H. (2008). Apoptotic effect of hesperidin through caspase3 activation in human colon cancer cells, SNU-C4. *Phytomedicine*.

[39] Lee H. Z., Leung H. W. C., Lai M. Y., Wu C. H. (2005). Baicalein induced cell cycle arrest and apoptosis in human lung squamous carcinoma CH27 cells. *Anticancer Research*.

[40] Lee K. W., Kim H. J., Lee Y. S. (2007). Acteoside inhibits human promyelocytic HL-60 leukaemia cell proliferation via inducing cell cycle arrest at G 0/G 1 phase and differentiation into monocyte. *Carcinogenesis*.

[41] Zhao L., Wientjes M. G., Au J. L. (2004). Evaluation of combination chemotherapy: integration of nonlinear regression, curve shift, isobologram, and combination index analyses. *Clinical Cancer Research*.

[42] Efferth T., Koch E. (2011). Complex interactions between phytochemicals. The multi-target therapeutic concept of phytotherapy. *Current Drug Targets*.

[43] Nessa M. U., Beale P., Chan C., Yu J. Q., Huq F. (2012). Studies on combination of platinum drugs cisplatin and oxaliplatin with phytochemicals anethole and curcumin in ovarian tumour models. *Anticancer Research*.

[44] Pinmai K., Chunlaratthanabhorn S., Ngamkitidechakul C., Soonthornchareon N., Hahnvajanawong C. (2008). Synergistic growth inhibitory effects of Phyllanthus emblica and Terminalia bellerica extracts with conventional cytotoxic agents: doxorubicin and cisplatin against human hepatocellular carcinoma and lung cancer cells. *World journal of gastroenterology: WJG*.

[45] Saab A. M., Tundis R., Loizzo M. R. (2012). Antioxidant and antiproliferative activity of Laurus nobilis L.(Lauraceae) leaves and seeds essential oils against K562 human chronic myelogenous leukaemia cells. *Natural Product Research*.

[46] Guerriero E., Sorice A., Capone F. (2017). Combining doxorubicin with a phenolic extract from flaxseed oil: evaluation of the effect on two breast cancer cell lines. *International Journal of Oncology*.

[47] Yue Q. X., Xie F. B., Guan S. H. (2008). Interaction of Ganoderma triterpenes with doxorubicin and proteomic characterization of the possible molecular targets of Ganoderma triterpenes. *Cancer Science*.

[48] Khan M. A., Singh M., Khan M. S., Najmi A. K., Ahmad S. (2014). Caspase mediated synergistic effect of Boswellia serrata extract in combination with doxorubicin against human hepatocellular carcinoma. *BioMed Research International*.

[49] Kumar H., Savaliya M., Biswas S. (2016). Assessment of the in vitro cytotoxicity and in vivo anti-tumour activity of the alcoholic stem bark extract/fractions of Mimusops elengi Linn. *Cytotechnology*.

[50] O'Brien P., Haskins J. R., Taylor D. L., Haskins J. R., Giuliano K. A. (2007). In vitro cytotoxicity assessment. *High Content Screening. Methods in Molecular Biology, vol 356*.

[51] Ifeoma O., Oluwakanyinsola S. (2013). Screening of herbal medicines for potential toxicities. *New Insights into Toxicity and Drug Testing*.

[52] ISO - The International Organization for Standardization (2009). *ISO 31000:2009 - Risk management - principles and guidelines, ISO 31000:2009*.

[53] Wang J., Kubes P. (2016). A reservoir of mature cavity macrophages that can rapidly invade visceral organs to affect tissue repair. *Cell*.

[54] Gul M. Z., Bhat M. Y., Maurya R., Qureshi I. A., Ghazi I. A. (2018). In vitro evaluation of antioxidant and antiproliferative activities of Artemisia nilagirica extracts. *Indian Journal of Pharmaceutical Sciences*.

[55] Girma S., Giday M., Erko B., Mamo H. (2015). Effect of crude leaf extract of Osyris quadripartita on Plasmodium berghei in Swiss albino mice. *BMC Complementary and Alternative Medicine*.

[56] Subba B., Srivastav C., Kandel R. C. (2016). Scientific validation of medicinal plants used by Yakkha community of Chanuwa VDC, Dhankuta, Nepal. *Springerplus*.

